# On the Use of Stannum

**Published:** 1810-04

**Authors:** 


### *On the USE of STANNUM.*

By Dr. Shearman.

It has been the lot of almost every substance employed as a remedy in the practice of medicine, to have its virtues at one time highly extolled, and to be very much over-rated, while, at another time, the same substance is depreciated in the public estimation as much below its real utility, and not unfrequently falls entirely into disuse, and is altogether neglected. Hence many articles of the Materia Medica are never, or very rarely employed, because they have not answered the extravagant encomiums bestowed upon them, by those who first discovered or introduced them, and a conclusion is too hastily drawn, that since they cannot effect every thing that has been boasted concerning them, they are unworthy to be retained in practice for the purposes they are capable of answering.

Stannum, in common with other substances, has undergone somewhat of a revolution of this kind; at no time, however, have its virtues been so extravagantly magnified, as we find to be the case with many others, while yet several beneficial effects have been formerly attributed to the employment of it in diseases, which at the present time, are altogether denied to it. There requires no further proof of this, than the variety of preparations of this metal, contained in the older Pharmacopœias, and the virtues attributed to each of them by the compilers of that day, contrasted with the slighting treatment it has met with in the two last Pharmacopœias of the British Colleges.

Without attempting to justify in the least, the high estimation in which it was formerly held, I cannot help thinking, but that the Edinburgh College, in giving no directions for preparing the powder, and the London College, by restricting the employment of it to its metallic filings, have not justly appreciated its effects upon the animal œconomy, and have deprived us of an useful remedy in some cases of diseases, which reluctantly yield to any medicines whatever, and which may sometimes be overcome by this metal, when none others are of any avail. That it is occasionally useful as an anthelmintic, every one seems willing to allow, and both Colleges appear to have proceeded upon the ground of its being useful ful for nothing else; both seem to have taken up the idea, that its action as an anthelmintic being purely mechanical, the form in which they have directed it, is the one best calculated to produce the effect expected from its employment in that view. That this accords with the general opinion of Physicians, I am led to believe, from the statement given by the learned Editor of the Edinburgh New Dispensatory, who, under the article Stannum, informs us, " that it is now only used as an anthelmintic, especially in cases of tænia," adding from himself, that " probably it acts mechanically."

A long use of this metal, however, has impressed me with an opinion, that its utility in cases of disease, is not confined to its mechanical action upon the intestines; since I have frequently found it serviceable in several cases of what are usually called *nervous affections*, more especially in head-achs that have been so denominated; many of which have yielded to it, when other remedies have failed, though its success has been somewhat uncertain, and at times has required a long perseverance in its exhibition to insure it, owing probably to its most effective preparation not having been always employed.

I well know that many affections of the head are symptomatic of irregular action in the alimentary canal, and are produced by diseases situated there; and our nosologists have not been sparing in arranging many convulsive diseases as being symptomatic of worms in the intestines, which would afford a presumption, that all the good effects experienced from the use of Stannum in such affections, may be attributed to its anthelmintic properties; but I suspect some fallacy in the opinion of worms being frequently a cause of these affections, they are themselves most commonly a symptom only of the unhealthy state of the alvine canal, and when this is corrected, the worms of course cease to exist, there being no longer a proper nidus for them. The good effects of this remedy, whatever they may be, are not increased by that state of the metal in which its mechanical powers are the greatest, but directly the contrary; and accordingly, I have always employed the metal in the finest state of powder in which I could procure it: this has generally been obtained by the process directed in the London Pharmacopœia of 1787, a very fine sieve being employed for the separation of the powder, which, even in this state, retains somewhat of a metallic appearance,

Two Cases have lately come under my observation, in which the use of tin has been followed by the most beneficial consequences, and which I could not fail to attribute to the minute degree of division the metal had undergone. I shall briefly relate them, as they appear recorded in my notes taken during the time the patients were under my care, at the Dispensary, in Old Gravel-lane.

Aaron Brown, aged 11 years, about five weeks before I saw him, had been attacked with head-ache and a considerable pain in his loins, loss of appetite and costiveness; three weeks after that, was seized with epileptic fits, recurring several times daily; they were always preceded by pain in his back, which seemed to extend up to his head; the integuments of the loins were tender to the touch, and there appeared a slight degree of swelling; he had not received any injury. I gave him 6 grains of calomel at night and a cathartic in the morning, and these were repeated at the interval of two days; they produced copious, slimy, and fœtid evacuations. The appetite now became keen; none of the other symptoms were relieved. A blister was applied to the loins, and a grain of calomel exhibited night and morning. The calomel was continued a fortnight, with cathartics occasionally interposed, within which time another blister was applied. No alteration in the symptoms, and the fits were as numerous and violent as ever.

*Nov.* 13. Stanni præparati ℈i. nocte et mane.

17. Symptoms all the same; bowels regular. Cont. stannum. empl. lyttæ ad lumbos.

20. Blister had risen well; he had several fits in the night of the 17th, and morning of the 18th, but has had none since. Upon being told the powders given him on the 17th were different from his former ones, I examined them; they had nothing of the dark metallic colour of the stannum, but consisted of a fine impalpable powder, nearly of the colour of fresh powdered ipecacuanha. Upon inquiry, I found, that he had been supplied from a fresh parcel of tin, sent in by a different druggist; and from him I learnt, that it had been prepared by subjecting the powdered tin, separated by a fine sieve, to levigation and elutriation,* in the manner directed by the Pharmacopœia for the preparation of chalk.

27. Had

* Does not this operation produce some considerable oxydation of the metal?

27. Had experienced no return of his fits. Dismissed cured.

The sudden amendment of this boy, immediately upon the exhibition of the new parcel of tin, struck me, as being curious, and I wished to ascertain what share the medicine had in producing this change. Happening to have under my care, at this very time, in the Dispensary, a favourable subject, I again exhibited it.

John Smith, aged 5 years, of dull faculties and fatuitous appearance, about nine weeks ago fell down upon the ground, since which time he has daily had several fits, of an epileptic form; previous to the fits, he complains of a pain in the abdomen, which feels rather tense and hard; they are succeeded by a stupor and profuse sweating; he sleeps well at night. The appetite was at first voracious, is now more moderate. He came under my care about three weeks ago, during which time, he has been taking calomel and cathartic medicines without much apparent benefit.

*Monday, Nov.* 27. Stanni præparati ℈ij. nocte & mane.

*Friday, Dec.* 1. Owing to some mistake of the person who fetched the medicine, he had taken only one dose, which was on Monday night; no return of the fits on Tuesday; two slight ones on Wednesday, but not any since. Repetatur Stannum.

This boy, who had all along been very punctual in his attendance, never once having missed, and whose mother was so very anxious for his recovery, as hitherto, implicitly to have obeyed all directions, came no more to the Dispensary, by which I am led to believe he had no further return of his fits, and neglected to come for a letter of thanks, a practice too common among dispensary patients when they no longer need assistance.

I too well know the fallacy of medical experience, to ground any conclusions upon these two cases, one of them rather imperfect indeed; but I think the coincidence sufficiently striking to merit further attention. In Brown's case, the fits returned no more after two doses of *fine tin* had been taken, though the former ones of coarsely powdered tin had produced no effect upon the disease. Smith, after a single dose, passed the whole of the next day without a fit, and the second day after that without any, although he had never missed them before. In neither of the cases were any worms voided; and in Brown, the discharge of fœtid slimy mucus did not prevent the accession of the fits. I shall not omit any future opportunity of giving this medicine; but the only way duly to appreciate the

 value value of any remedy is, to make a great many trials by several different practitioners; and those who can readily believe that the same substance, under different states of preparation, may produce very various effects upon the animal system, will perhaps be induced to subject this question to the test of their experience.

